# Optimal Frailty Dimensions for Assessing Frailty and Predicting Chemotherapy Adverse Events in Older Taiwanese Cancer Patients

**DOI:** 10.3389/fmed.2022.828865

**Published:** 2022-06-14

**Authors:** Ya-Wen Ho, Shih-Ying Chen, Yu-Shin Hung, Shinn-Yn Lin, Wen-Chi Chou

**Affiliations:** ^1^Division of Hematology-Oncology, Department of Internal Medicine, Chang Gung Memorial Hospital at Linkou, Taoyuan, Taiwan; ^2^School of Nursing, College of Medicine, Chang Gung University, Taoyuan, Taiwan; ^3^College of Medicine, Chang Gung University, Taoyuan, Taiwan; ^4^Department of Radiation Oncology, Chang Gung Memorial Hospital at Linkou, Taoyuan, Taiwan

**Keywords:** geriatric assessment, frailty, frail dimension, chemotherapy adverse events, elderly cancer patients

## Abstract

**Background:**

This study aimed to investigate the effects of different frailty dimensions on frailty prevalence in older Taiwanese cancer patients receiving chemotherapy, and to analyze the dimensions that should be included in frailty assessment for effectively predicting serious adverse events, unexpected hospitalizations, and emergency department visits.

**Materials and Methods:**

This study prospectively enrolled 234 cancer patients with solid cancer or lymphoma and aged 65 years or older who later received chemotherapy at a medical center in Taiwan from September 2016 to November 2018. First, all patients were subjected to a frailty assessment on eight frailty dimensions within 1 week before their first chemotherapy treatment. The effects of different dimensions on frailty were analyzed using a Poisson regression model. Second, after sequentially excluding one, two, and three dimensions with the lowest effects, frailty was sequentially assessed in the remaining seven, six, and five dimensions for comparison of chemotherapy-related adverse events.

**Results:**

Nutritional status, comorbidity, history of falls, cognitive status, and polypharmacy were the top five important dimensions of frailty in older Taiwanese cancer patients. Regardless of the number (five to eight) of dimensions used for frailty assessment, frail patients had higher rates of serious adverse events, unexpected hospitalizations, and emergency room visits than non-frail patients during chemotherapy.

**Conclusions:**

Frailty assessment in older Taiwanese cancer patients should be based on at least five dimensions to accurately identify those at high risk of serious adverse events during chemotherapy. It is expected that the present findings may be used to design a frailty scale for older Taiwanese in the future.

## Introduction

Frailty refers to a decrease in the reserve capacity of multiple systems of the body, which increases the risk of adverse health outcomes when stressful events occur and the body is unable to respond (1, 2). The gradual degeneration of physiological systems with age combined with chronic comorbidities, nutritional deficiencies, and reduced family social support put the older (age ≥ 65) at high risk of developing frailty (3–5). For patients with cancer, the cancer itself and related anticancer treatments accelerate the decline of physiological reserve capacity (1), making older cancer patients a high-risk group for developing frailty (6).

Frailty has recently drawn great attention in medical research on older cancer patients. Studies have shown that frailty can be used to predict the tolerability of chemotherapy (1, 6), chemotherapy-related side effects (7, 8), treatment discontinuation (4), emergency department visits or hospitalization (4), and even death (3, 7, 9) in older cancer patients. Therefore, the American Society of Clinical Oncology (ASCO), the National Comprehensive Cancer Network (NCCN), and the International Society of Geriatric Oncology (ISGO) strongly recommend that before receiving cancer treatment, older patients should receive a multi-dimensional geriatric assessment (GA) to identify the frailty of older cancer patients or the geriatric issues that have not been identified via routine assessments (10–12). It is recommended that a comprehensive frailty assessment should involve the following eight dimensions: functional status, cognitive status, nutritional status, psychological mood, social support, polypharmacy, chronic comorbidity, and history of falls (10–13). Most scholars define frailty as the exhibition of deficits in two or more dimensions (6, 14). However, different international guidelines have inconsistent recommendations for frailty assessment. For example, ASCO/SIOG (10, 12) recommends that physical assessment should include the following dimensions: Function, Cognition, Nutrition, Depression, Comorbidity, and Falls, while NCCN (11) recommends that Socioeconomic and Polypharmacy should also be included in the assessment, but these guidelines do not emphasize which dimensions are the most essential for frailty assessment in cancer patients, nor do they mention how many dimensions are needed to constitute a frailty assessment tool. More importantly, the above guidelines all target Western populations. Research on the frailty of older Taiwanese cancer patients is in its infancy, and it is not yet clear which physical and mental deficits are the most important factors associated with the frailty of older Taiwanese cancer patients. The present study aimed to explore the effects of physical and mental deficits in different frailty dimensions on frailty prevalence in older Taiwanese cancer patients, and to identify the minimum number of dimensions to be included in constituting an appropriate frailty assessment tool to predict serious adverse events in older Taiwanese cancer patients receiving chemotherapy.

## Patients and Methods

### Study Population

This study was part of a prospective, longitudinal and observational study conducted to evaluate the impact of frailty on treatment outcome in cancer patients in Taiwan (15). The study was carried out at a medical center in northern Taiwan from August 2016 to June 2018. The inclusion criteria were: patients (1) ≥65 years of age or older; (2) pathologically diagnosed with solid cancer or lymphoma who, within 1 week of the diagnosis, were expected to start adjuvant chemotherapy for solid cancer or receive cyclophosphamide, hydroxydaunorubicin, oncovin, and prednisone (CHOP)-like chemotherapy for lymphoma (16); (3) who were conscious and able to communicate verbally or in writing; and (4) who signed the respondent's consent form. Patients who were to receive chemotherapy for non-curative purposes, receive synchrotron radiation therapy, or were unable to sign the respondent's consent form were excluded.

### Data Collection

All patients underwent a multidimensional frailty assessment by trained research assistants within 1 week prior to the anticipated first chemotherapy treatment. Meanwhile, the following basic information was collected from the patients: sex, age, marital status, education, work status, primary caregiver, cancer type, cancer stage, chemotherapy drugs, Eastern Cooperative Oncology Group (ECOG) performance status (PS), body mass index (BMI), chronic disease, smoking history, and alcohol consumption. The types and formulations of chemotherapy drugs were decided by physicians according to the institution's guidelines of personalized cancer treatment.

During chemotherapy, patients returned to the medical center every two to three weeks for treatment and evaluation of adverse events. All chemotherapy-related serious adverse events, unexpected hospitalizations or emergency department visits were noted, from the first day of chemotherapy to 3 months after the initiation of therapy. A serious adverse event was defined as developing ≥ grade 3 chemotherapy toxicity according to CTCAE Version 4.0 of the National Cancer Institute (17).

### Frailty Assessment

Frailty was assessed on eight dimensions: functional status,cognition, nutrition, psychological mood, social support, polypharmacy, comorbidity, and history of falls. The detail tool and cutoff standard for each frail condition used in this study has been validated in Taiwanese older cancer patients according to our previous study (15). Frailty was defined as showing deficits in two or more dimensions according to cancer patient-related frailty literature (6, 14). and Taiwan-based studies on frailty in cancer patients (15, 18, 19). and the older general population (20).

### Data Analysis

A two-tailed statistical analysis was performed using Statistical Package SPSS 20.0 for Windows, with a *p*-value < 0.05 considered indicative of statistical significance. The effects of physical and mental deficits in eight frailty dimensions on frailty prevalence were evaluated using a Poisson regression model, aiming to reveal the relative importance of each frailty dimension in predicting the responses of older Taiwanese cancer patients to treatment. Poisson regression is basically a count regression approach; it is suitable for binary variables if the outcome is rare (21). Firstly, all of the eight dimensions were used to assess the patient's frailty. According to the effect (as measured by the beta coefficient of the Poission regression model) of a deficit in a given frailty dimension on frailty prevalence, the dimensions with the smallest deficit effects (social support, functional status, and psychological mood) were excluded sequentially in order to perform a seven, six, and five-dimensional frailty assessment as well, as an association analysis between frailty and chemotherapy adverse events, totaling four analyses. In addition, a chi-square test was conducted to evaluate the association of the frailty assessed in eight, seven, six, or five dimensions with the incidence of serious adverse events, unexpected hospitalizations, and emergency department visits within 3 months after the initiation of chemotherapy treatment.

## Results

### Patient Basic Information

A total of 234 older cancer patients was enrolled. The median age was 70 years (range 65–96 years), and most patients were female (53%) and married (80.8%). Lymphoma (32.5%) was the most prevalent cancer, followed by breast cancer (22.2%) and colorectal cancer (20.1%). The majority of patients had stage II (34.2%) and stage III cancers (40.2%) and were treated with multiple chemotherapy regimens (82.9%), with ECOG PS scores of 0 (58.1%) and 1 (36.8%) (Table 1).

**Table 1:** Basic information of older cancer patients (*N* = 234).

**Variable**	**Number**	**%**
**Gender**		
Female	124	53
Male	110	47
**Age, years**	Median: 70	(Range: 65–96)
65−69	103	44
70–74	67	28.6
75–79	45	19.2
≧80	19	8.2
**Marriage**		
Married	189	80.8
Others	45	19.2
**Education**		
Junior high school or less	145	62
Senior high school or more	89	38
**Occupation**		
No	203	86.8
Yes	31	13.2
**Main caregiver**		
Spouse	121	51.7
Child	87	37.2
Others	26	11.1
**Cancer type**		
Lymphoma	76	32.5
Breast	52	22.2
Colorectal	47	20.1
Gastrointestinal	27	11.5
Lung	12	5.1
Urological	10	4.3
Others*	10	4.3
**Stage**		
I	20	8.5
II	80	34.2
III	94	40.2
IV	40	17.1
**Chemotherapy regimen**		
Monotherapy	40	17.1
Combination therapy	194	82.9
**ECOG**		
0	136	58.1
1	86	36.8
≧2	12	5.2
**BMI, kg/m** ^**2**^	Median: 23.5	(Range: 16–42.4)
<19	18	7.7
19 – <21	30	12.8
21 – <23	53	22.6
≧23	133	56.8
**Comorbidity**		
No	67	28.6
Yes	167	71.4
**Smoking**		
No	155	66.2
Yes	79	33.8
**Drinking**		
No	165	70.5
Yes	69	29.5

^*^head & neck (6), gynecologic (3), cancer of unknown primary cause (1).

### Frailty Dimensions Commonly Included in a Frailty Assessment and the Corresponding Prevalence of Frailty

Among the 234 patients, frailty deficits occurred most frequently in the nutritional status dimensio*n* (65.4%), followed by comorbidity (38.5%), functional status (24.8%), polypharmacy (23.1%), psychological mood (17.5%), history of falls, (13.2%), cognitive status (10.7%), and social support (9.4%) (Table 2). Among the 234 patients, 40 (17.1%), 58 (24.8%), 52 (22.2%), 42 (17.9%), 28 (12%), and 14 (6%) had 0, 1, 2, 3, 4, and ≧5 physical and mental deficits, respectively. The prevalence of frailty was 58.1% when using all eight dimensions of physical and mental deficits for the frailty assessment and decreased to 56.0% when the social support dimension was excluded, and the remaining seven dimensions were used for the assessment. The prevalence of frailty further decreased to 52.1% when both social support and functional status were excluded and the assessment was based on the remaining six dimensions. When the social support, functional status, and psychological mood dimensions were excluded, the frailty assessed in the remaining five dimensions had a prevalence of 47.4%.

**Table 2:** Defect distribution in multiple frailty dimensions of older cancer patients (*N* = 234).

**Frailty dimension**	**Measures**	**Number** **of items**	**Score Range**	**Cutoff** **value**	***N* (%)**
Functional status	ADL IADL ADL or IADL	10 8	0–100 0–8	<100 <8	46 (19.7) 37 (15.8) 58 (24.8)
Cognition	Modified short version MMSE	13	0–13	<9	25 (10.7)
Nutrition	MNA-SF	6	0–8	<12	153 (65.4)
Mood	GDS-4	4	0–4	>1	41 (17.5)
Social support	Living alone or lack of family support	1	Yes/No	Yes	22 (9.4)
Polypharmacy	Number of medications	1	0–∞	>4	54 (23.1)
Comorbidity	CCI	19	0–33	>1	90 (38.5)
Mobility/Falls	Number of falls	1	0–∞	>1	31 (13.2)

*ADL, activities of daily living; CCI, charlson comorbidity index; GDS, geriatric depression scale; IADL, instrumental activities of daily living; MNA-SF, mini nutritional assessment-short form*.

### Effect of a Deficits in Various Frailty Dimensions on Frailty Prevalence

As revealed by the Poisson regression model, the effect of a deficit in various frailty dimensions on frailty prevalence in older cancer patients could be ranked in order of the decreasing beta coefficient: nutritional status (β = 0.99, odds ratio [OR]. = 2.70, 95% CI 1.61 – 4.53), comorbidity (β = 0.58, OR = 1.79, 95% CI 1.25 – 2.55), history of falls (β = 0.51, OR = 1.66, 95% CI 1.08 – 2.56), cognitive status (β = 0.41, OR = 1.51, 95% CI 0.91 – 2.51), polypharmacy (β = 0.30, OR = 1.34, 95% CI 0.93 – 1.95), psychological mood (β = 0.27, OR = 1.31, 95% CI 0.88 – 1.93), functional status (β = 0.26, OR = 1.29, 95% CI 0.83 – 2.02), and social support (β = 0.11, OR = 1.12, 95% CI 0.66 – 1.91) (Table 3).

**Table 3:** Effects of physical and mental deficits in different frailty dimensions on frailty prevalence in older cancer patients (*N* = 234) using the Poisson regression analysis.

**Frailty dimension**	**Beta coefficient**	**Standard error**	**Odds ratio (95% confidence interval)**
Nutrition	0.99	0.26	2.70 (1.61 – 4.53)
Comorbidity	0.58	0.18	1.79 (1.25 – 2.55)
Mobility/Fall	0.51	0.22	1.66 (1.08 – 2.56)
Cognition	0.41	0.26	1.51 (0.91 – 2.51)
Polypharmacy	0.30	0.19	1.34 (0.93 – 1.95)
Psychological mood	0.27	0.20	1.31 (0.88 – 1.93)
Function_ADL	0.26	0.23	1.29 (0.83 – 2.02)
Social support	0.11	0.27	1.12 (0.66 – 1.91)
Function_IADL	0.07	0.26	0.93 (0.56 – 1.54)

*ADL, activities of daily living; IADL, instrumental activities of daily living*.

### Frailty and Chemotherapy Adverse Events

Table 4 presents the association of frailty, assessed on a different number of frailty dimensions, with chemotherapy adverse events. When frailty was assessed using physical and mental deficits in eight dimensions, there were significant differences between the frailty group and the non-frailty group in the incidence of grade 3–4 toxicity, namely in the incidence of thrombocytopenia (1.0 vs. 10.3%, *p* = 0.005), non-hematologic toxicity (25.5 vs. 39.0%, *p* = 0.035), and hyponatremia (1.0 vs. 11.0%, *p* = 0.003).

**Table 4:** Association of the frailty assessed in a different number of dimensions to serious chemotherapy adverse events in older cancer patients (*N* = 234).

**Serious adverse event**	**8 domains**			**7 domains**			**6 domains**			**5 domains**		
	**Non-frailty (*n =* 98)**	**Frailty** **(*n =* 136)**		**Non-frailty** **(*n =* 103)**	**Frailty** **(*n =* 131)**		**Non-frailty** **(*n =* 112)**	**Frailty** **(*n =* 122)**		**Non-frailty** **(*n =* 123)**	**Frailty** **(*n =* 111)**	
	***n* (%)**	***n* (%)**	***p***	***n* (%)**	***n* (%)**	***p***	***n* (%)**	***n* (%)**	***p***	***n* (%)**	***n* (%)**	***p***
**Any hematological**	34 (34.7)	54 (39.7)	0.495	35 (34.0)	53 (40.5)	0.34	40 (35.7)	48 (39.3)	0.59	43 (35.0)	45 (40.5)	0.42
Low hemoglobin	7 (7.1)	20 (14.7)	0.097	8 (7.8)	19 (14.5)	0.15	10 (8.9)	17 (13.9)	0.31	11 (8.9)	16 (14.4)	0.22
Thrombocytopenia	1 (1.0)	14 (10.3)	**0.005**	1 (1.0)	14 (10.7)	**0.002**	2 (1.8)	13 (10.7)	**0.006**	2 (1.6)	13 (11.7)	**0.002**
Leukopenia	16 (16.3)	29 (21.3)	0.40	17 (16.5)	28 (21.4)	0.41	20 (17.9)	25 (20.5)	0.62	22 (17.9)	23 (20.7)	0.62
Neutropenia	29 (29.6)	45 (33.1)	0.67	30 (29.1)	44 (33.6)	0.48	35 (31.2)	39 (32.0)	0.99	38 (30.9)	36 (32.4)	0.89
Neutropenia with fever	5 (5.1)	15 (11.0)	0.15	5 (4.9)	15 (11.5)	0.10	6 (5.4)	14 (11.5)	0.11	7 (5.7)	13 (11.7)	0.11
**Any non-hematological**	25 (25.5)	53 (39.0)	**0.035**	25 (24.3)	53 (40.5)	**0.01**	31 (27.7)	47 (38.5)	0.10	32 (26.0)	46 (41.4)	**0.02**
Hyponatremia	1 (1.0)	15 (11.0)	**0.003**	1 (1.0)	15 (11.5)	**0.001**	3 (2.7)	13 (10.7)	**0.02**	4 (3.3)	12 (10.8)	**0.04**
Hypokalemia	4 (4.1)	10 (7.4)	0.405	4 (3.9)	10 (7.6)	0.28	6 (5.4)	8 (6.6)	0.79	6 (4.9)	8 (7.2)	0.58
Hyperglycemia	4 (4.1)	12 (8.8)	0.195	4 (3.9)	12 (9.2)	0.13	4 (3.6)	12 (9.8)	0.07	4 (3.3)	12 (10.8)	**0.04**
Infection	10 (10.2)	26 (19.1)	0.068	10 (9.7)	26 (19.8)	**0.04**	14 (12.5)	22 (18.0)	0.28	14 (11.4)	22 (19.8)	0.10
Hypertension	14 (14.3)	19 (14)	0.99	14 (13.6)	19 (14.5)	0.99	17 (15.2)	16 (13.1)	0.71	17 (13.8)	16 (14.4)	0.99
**Unexpected hospitalizations**	15 (15.3)	38 (27.9)	**0.027**	16 (15.5)	37 (28.2)	**0.027**	21 (18.8)	32 (26.2)	0.211	24 (19.5)	29 (26.1)	0.274
**Emergency department visit**	16 (16.3)	38 (27.9)	**0.042**	17 (16.5)	37 (28.2)	**0.042**	22 (19.6)	32 (26.2)	0.278	24 (19.5)	30 (27.0)	0.214

*For chemotherapy-related adverse outcomes, grade 3–4 toxicity occurs in 5% or more of patients before being shown in the table above*.

*8 domains: Nutrition/ Comorbidity/ Falls/ Cognition/ Polypharmacy / Mood/ Functional status/ Social support*.

*7 domains: Nutrition/ Comorbidity/ Falls/ Cognition/ Polypharmacy / Mood/ Functional status*.

*6 domains: Nutrition/ Comorbidity/ Falls/ Cognition/ Polypharmacy / Mood*.

*5 domains: Nutrition/ Comorbidity/ Falls/ Cognition/ Polypharmacy*.

*The bold value indicates existence of a statistically significant difference with p < 0.05*.

When frailty was assessed using physical and mental deficits in seven frailty dimensions, there were significant differences between the frailty group and the non-frailty group in the incidence of grade 3–4 toxicity, namely in the incidence of thrombocytopenia (1.0 vs. 10.7%, *p* = 0.002), non-hematologic toxicity (24.3 vs. 40.5%, *p* = 0.01), hyponatremia (1.0 vs. 11.5%, *p* = 0.001), and infectio*n* (9.7 vs. 19.8%, *p* = 0.04).

When frailty was assessed using physical and mental deficits in six frailty dimensions, there were significant differences between the frailty group and the non-frailty group in the incidence of grade 3–4 toxicity, namely in the incidence of thrombocytopenia (1.8 vs. 10.7%, *p* = 0.006) and hyponatremia (2.7 vs. 10.7%, *p* = 0.02).

When frailty was assessed using physical and mental deficits in 5 frailty dimensions, there were significant differences between the frailty group and the non-frailty group in the incidence of grade 3–4 toxicity, namely in the incidence of thrombocytopenia (1.6 vs. 11.7%, *p* = 0.002), non-hematologic toxicity (26.0 vs. 41.4%, *p* = 0.02), hyponatremia (3.3 vs. 10.8%, *p* = 0.04), and hyperglycemia (3.3 vs. 10.8%, *p* = 0.04).

### Association of Frailty With Unexpected Hospitalizations and Emergency Department Visits

A total of 53 (22.6%) and 54 (23.1%) of the 234 patients experienced unexpected hospitalizations and emergency department visits during treatment. When frailty was assessed using physical and mental deficits in eight frailty dimensions, the frailty group had a higher incidence of unexpected hospitalizations (27.9 vs. 15.3%, *p* = 0.027) and emergency department visits (27.9 vs. 16.3%, *p* = 0.042) than the non-frailty group (Table 4). The frailty group still had a higher incidence of unexpected hospitalizations (28.2 vs. 15.5%, *p* = 0.027) and emergency department visits (28.2 vs. 16.5%, *p* = 0.042) than the non-frailty group when frailty was assessed in seven dimensions. However, when frailty was assessed in six or five dimensions, the between-group differences in the incidence of unexpected hospitalizations and emergency department visits were not statistically significant, although the incidence was higher in the frailty group.

## Discussion

Comprehensive frailty assessment is currently the widely accepted gold standard for assessing frailty, but there is still no international consensus with regard to what and how many dimensions of physical and mental deficits should be included for appropriate frailty assessment (10–12). The present study revealed that nutritional status, comorbidity, history of falls, cognitive status, and polypharmacy were the top five important dimensions of frailty in older Taiwanese cancer patients. In addition, the results showed that frailty was a common condition among older Taiwanese cancer patients, with 58% of patients experiencing frailty when frailty was assessed using eight dimensions, and still 47% when frailty was assessed using only five dimensions. Regardless of the number (five to eight) of dimensions used for frailty assessment, frail patients had higher rates of serious adverse events, unexpected hospitalizations, and emergency room visits than non-frail patients during chemotherapy. Therefore, each older Taiwanese cancer patient should be routinely assessed for frailty prior to chemotherapy, in order to facilitate early identification of those at high risk for serious adverse events, so that interventions for reducing the incidence of adverse events and improving prognosis may be performed.

A review article of 20 studies on non-Taiwanese populations showed that the median prevalence of frailty in older cancer patients is 43% (range 7–68%), (6). while the incidence in older Taiwanese cancer patients has been reported to be 28.9–47.6% (3, 18–22). The prevalence of frailty in the present study was higher than that in other studies. It is noteworthy that most of the studies included in the review article above classified patients into three groups, (6). namely a non-frailty group, a pre-frailty group, and a frailty group, rather than the two groups (frailty vs. non-frailty) used in the present study. The likely misclassification of frail subjects as pre-frailty subjects may explain the lower prevalence of frailty in non-Taiwanese cancer patients compared with the present study. Among studies on the frailty of Taiwanese cancer patients, a study by Chou et al. showed that the prevalence of frailty in older patients with hematologic cancers was 35.5% (3). However, that study assessed frailty in terms of physical and mental deficits in seven frailty dimensions, and it defined frailty as showing abnormality in three or more dimensions. Another study on patients with head and neck cancer showed that the prevalence of frailty in the patients was 28.9% (frailty was assessed using seven dimensions and was defined as showing abnormality in ≧2 dimensions), (19). but the enrolled subjects had relatively low ages (median age 54 years, range 24–86 years), which resulted in a much lower prevalence of frailty compared with the present study. Chen et al. used physical and mental deficits in five dimensions to assess frailty and reported a frailty prevalence of 47.6% in older cancer patients, (18). a result almost identical to the frailty prevalence observed in the present study using the same number of dimensions. As is the case with Taiwan-based frailty surveys for cancer patients, (3, 18, 19). the present study revealed a very high prevalence of frailty in Taiwanese cancer patients. Our results indicate the importance of a frailty assessment of local older cancer patients.

Nutritional status was the most important dimension of frailty in older cancer individuals in the present study, with up to 65.4% of patients suffering from malnutrition. Malnutrition in cancer patients is caused by multiple factors, including the direct pressing of tumors on the gastrointestinal tract and chronic morbidities, which lead to nutrient loss or malabsorption. In addition, decline in physical fitness, insufficient cognitive function, and lack of social support can directly or indirectly lead to nutrient deficiencies (23). Therefore, an assessment of the nutritional status of older patients must take into account the presence or absence of functional impairment at multiple functional levels, and these considerations should constitute the basis of frailty assessment.

A retrospective study examining the nutritional status of cancer patients showed that the prevalence of nutritional deficiency before chemotherapy is 1/3 or higher in all cancer patients (24). and is as high as 44.6% in older cancer patients (25). Malnutrition is a poor prognostic factor for cancer patients: it not only increases mortality, but also the incidence of adverse effects associated with anticancer treatment, causes treatment discontinuation, and affects patients' quality of life (26). In Taiwan, 47% of head and neck cancer patients have malnutrition before concurrent chemoradiotherapy (CCRT), and 20% of these malnourished patients do not even receive nutritional counseling (20), while for the other 53% of patients with normal nutritional status, nutritional counseling before CCRT may significantly improve survival and reduce the discontinuation rate of CCRT (27). The present study shows that a nutritional assessment is extremely important for cancer patients receiving anti-cancer treatment. In addition to routine weight monitoring, health care teams can provide early nutrition care, including nutrition-related health education or dietitian consultation, with the goal of not only correcting malnutrition but also of reducing frailty to improve the prognosis and reduce the side effects of cancer treatment (27, 28).

For cultural reasons, up to 87% of older Taiwanese live with their family members, (29). who take care of them and assist them in their daily lives. This was reflected in the present study results. Patients who lived alone or who lacked family support accounted for the smallest proportion of the 234 patients, at only 9.4%, thereby explaining why a deficit in the social support dimension would have the smallest effect on frailty prevalence in older Taiwanese cancer patients.

Previous studies have shown that a deficit in the functional status dimension is associated with chemotherapy grade 3 or higher toxicity (30, 31). However, the present study showed that the effect of a deficit in the functional status dimension on frailty prevalence was the second smallest, among all the types of deficits. The Lawton instrumental activities of daily living (IADL) scale used in the present study may not be applicable to older Taiwanese individuals. Most older Taiwanese live with their family members, (29). who take care of them in several aspects of their daily lives. Therefore, a high proportion of the present survey participants answered, “not applicable” to Lawton IADL items such as “food preparation,” “household maintenance” and “laundry” (36, 27, and 47%, respectively). In the Lawton IADL scale, the answer “not applicable” is given a full score, thus leading to underestimation of the effect of a deficit in the physical status dimension on frailty prevalence in older Taiwanese cancer patients. Therefore, it is recommended that the Lawton IADL scale should be used with caution when assessing the functional status of older patients in social groups with high family functioning, or when a patient answers “not applicable”. In the latter scenario, it is necessary to further confirm whether this answer is selected by the family members on behalf of the patient or is selected for other reasons, so as to further clarify the actual physical status of the patient. In addition, compared with patients who receive palliative chemotherapy, the patients enrolled in the present study were all to receive chemotherapy for the purpose of cure and, therefore, needed to meet more rigorous clinical requirements for physical fitness, so as to withstand the high intensity of the anti-cancer treatment. Accordingly, the proportion of patients with ECOG scores of 0–1 was high, at 95% in the present study. Due to such a high proportion of patients in a good physical condition, the effect of a deficit in the functional status dimension on frailty prevalence was underestimated.

The present results showed that there was a significant association between frailty and unanticipated hospitalizations/emergency department visits when frailty was assessed in either seven or eight dimensions, but the association was absent when frailty was assessed in either five or six dimensions. These observations suggest that the more frailty dimensions assessed, the more representative the assessment results are for the patients. However, a higher number of frailty dimensions assessed would make clinical practice more time-consuming, possibly reducing the actual use of the frailty assessment. To construct an efficient assessment tool using the minimum number of assessment factors, we propose that the frailty assessment of older Taiwanese cancer patients should be conducted using at least five dimensions, including at least nutritional status, comorbidity, history of falls, cognitive status, and polypharmacy.

The present study was the first of its kind to investigate the effects of physical and mental deficits in various frailty dimensions on frailty prevalence in older Taiwanese cancer patients. It provides information on the epidemiology of local frailty in older cancer patients and the functional deficits that commonly accompany frailty. It is expected that these findings may be used in the future to design a frailty scale for the Taiwanese population. However, the present study has some limitations. First, the subjects developed various types of cancers, among which the cycle and duration of chemotherapy, as well as the cumulative toxicity of chemotherapy formulations, may vary widely. To reduce these interferences, we collected the information of serious adverse events, unanticipated hospitalizations, and emergency department visits of all patients within the same period, namely within 3 months of the initiation of chemotherapy. Therefore, the association between frailty and long-term side effects of chemotherapy could not be examined in the present study. Furthermore, the association between frailty and chemotherapy-related adverse events remained in univariate analysis in our study. Statistical bias might exist because some potential confounding variables, including tumor type, tumor stage, and chemotherapy regimen, did not adjust for analysis. Second, the enrolled patients were those expected to receive curative chemotherapy. Such patients must be in good physical condition to withstand high-intensity therapy. Older patients in poor physical condition are considered to be unsuitable for curative chemotherapy. Thus this limits the generalizability of the present results. Third, this manuscript aimed to evaluate whether which frailty dimension was most importantly relevant to predict chemotherapy adverse events in order Taiwanese cancer patients. As a result, the impact of each frailty dimension on mortality, physical function, quality of life, body weight loss, and caregiver stress is not able to analyze in this study. In addition, The association of frailty and adverse events of chemotherapy remain analyzed in univariate model without considering the other confounding factors, including cancer types and intensity of chemotherapy. Fourth, the beta coefficient, but not Akaike's Information Criteria value, is used for dropping variables in the model section. Some significant variables with the smallest beta coefficient might exclude from analysis in our study. Finally, regardless of whether five to eight physical dimensions were used to assess patient frailty, frailty was defined as having functional deficits in ≥ 2 dimensions, which is a frailty criterion commonly used in countries and regions other than Taiwan. However, there is still a lack of Taiwan-based literature to confirm the applicability of this cut-off criterion to older Taiwanese cancer patients. Future research needs to explore a frailty cut-off criterion suitable for the Taiwanese population so as to establish a more complete frailty assessment tool for older cancer patients.

## Conclusions

The present results showed that nutritional status, comorbidity, history of falls, cognitive status, and polypharmacy were the top five most important dimensions of frailty in older Taiwanese cancer patients. The frailty scale, which includes these five dimensions, can be used to identify serious adverse events in local older cancer patients receiving high-intensity chemotherapy. It is expected that the present findings may be used to design a frailty scale for older Taiwanese in the future.

## Data Availability Statement

The original contributions presented in the study are included in the article/supplementary material, further inquiries can be directed to the corresponding author.

## Ethics Statement

The studies involving human participants were reviewed and approved by Chang Gung Memorial Hospital at Linkou. The patients/participants provided their written informed consent to participate in this study.

## Author Contributions

Y-WH, S-YC, and W-CC: conception and design of study. Y-WH and S-YC: acquisition of data. Y-WH, S-YC, and Y-SH: analysis and interpretation of data. Y-WH, S-YC, Y-SH, and W-CC: drafting of the manuscript. All authors contributed to the article and approved the submitted version.

## Funding

This study was supported by research grant (CMRPG3L1611) from Chang Gung Memorial Hospital at Linkou, Taoyuan, Taiwan, R.O.C. and research grant (PMRPG3M0031) from Ministry of Health and Welfare, Taiwan, R.O.C.

## Conflict of Interest

The authors declare that the research was conducted in the absence of any commercial or financial relationships that could be construed as a potential conflict of interest.

## Publisher's Note

All claims expressed in this article are solely those of the authors and do not necessarily represent those of their affiliated organizations, or those of the publisher, the editors and the reviewers. Any product that may be evaluated in this article, or claim that may be made by its manufacturer, is not guaranteed or endorsed by the publisher.
